# Supplementary material for: Genetic markers of abdominal obesity and weight loss after gastric bypass surgery
Source: PLoS One. 2021 May 28;16(5):e0252525. doi: 10.1371/journal.pone.0252525 (PMC8162622; doi:10.1371/journal.pone.0252525)
Supplement: S1 Table — (DOCX) [file pone.0252525.s001.docx]

Supplemental Table 1: Associations between baseline anthropometrics and weight loss after bariatric surgery

|  | β | 95% CI | p |
| --- | --- | --- | --- |
| Whole group | |  |  |
| BMI | 1.22 | 1.03 to 1.41 | <0.001 |
| WHR_BMI_ | -11.8 | -23.3 to -0.3 | 0.047 |
|  |  |  |  |
| Women alone | |  |  |
| BMI | 1.17 | 0.96 to 1.38 | <0.001 |
| WHR_BMI_ | -35.6 | -53.1 to -18.1 | <0.001 |
|  |  |  |  |
| Men alone | |  |  |
| BMI | 1.31 | 0.91 to 1.70 | <0.001 |
| WHR_BMI_ | -4.1 | -40.0 to 31.8 | 0.82 |

BMI; Body mass index. CI; Confidence interval. WHR; Waist-hip-ratio. WHR_BMI_; Waist-hip-ratio adjusted for BMI.

Linear regression with weight change as dependent variable (Δkg; n=576 for BMI; n=272 for WHR). The presented β values are effects per kg/m^2^ (BMI) or per WHR-unit (WHR_BMI_).
